# On Certain Conditions of Dead Teeth

**Published:** 1883-03

**Authors:** C. S. Tomes


### THE DENTAL REGISTER.

VOL. XXXVII.] MARCH, 1883. [No. 3.

### COMMUNICATIONS.

### On Certain Conditions of Dead Teeth.

BY. C. S. TOMES.

[Read before the Odontological Society of Great Britain.]

The treatment of dead teeth is, in the hands of most of us, at best uncertain; and although there are to be found practitioners who in all sincerity aver that they obtain perfectly satisfactory results with them, nevertheless when one finds these same persons spending much time and trouble over the confessedly uncertain operations of capping the pulp, etc., one can not but think that some form of enthusiasm has blunted the dispassionate scientific accuracy of their observing powers. The occurrence of one or two instructive failures in my own practice has suggested that, although I have nothing new to put forward, it would be worth while to take stock of our actual solid knowledge upon the subject, so as to realize what is fact and what is conjecture, and thus have a better standpoint from which to speculate upon the causes of non-success.

The problem is how best to get the organism to tolerate a partially devitalized body in continuity with it. To realize the nature of the problem we may fairly look for light to the structure of healthy teeth, and not in man alone; and also to any fair analogies that can be drawn from other parts of the body.

The most highly-organized parts of the body, those most alive, so to speak, are always protected by being buried away in its interior; external parts, subject to the chances of contact with the outside world, are protected by tissues less fully alive. Thus we are coated over with an epithelium, the deep portions of which are active cells, soft and full of plasm: the superficial layers are horny and all but dead, and as they become quite dead, or perhaps after in their dead condition they have remained a short time adherent, they are shed off. Other instances might be adduced, but this one will suffice to indicate the meaning, which is this: our highly organized live bodies are made fit to encounter the world by being coated with successive layers of less highly organized tissues, till at last in the outermost epithelium we come to something practically dead. This is tolerated because it is gradually led up to, but after all it is not very permanent and is constantly being cast off and renewed. I can not stop to point out how true it is to say that teeth are but skin appendages; but will simply point out that in their outer epithelial layer, the enamel, they are all but dead; that this nearly dead tissue is brought into continuity with the actively live organism by the intervention (i.) of dentine with its tooth-pulp, and (ii.) cementum with its alveolo-dentar periosteum; in other words, in the case of a perfect tooth and its surroundings very highly organized tissues are not asked to tolerate in contact with them that which is practically dead, without the intervention of other tissues less highly organized, so as to bridge over the abruptness of the change.

Comparative anatomy furnishes us with countless examples of teeth which have not a cementum with a highly organized periosteum, and in which the pulp (after once the tooth is formed) retains little or no vascularity nor functional activity. Such teeth are, in their entirety, like human enamel, practically dead; but like the similar external layers of epithelium of the skin, they are constantly being cast off and renewed; one might say of them that they were not tolerated long in continuity with the living body.

One of the ways in which dead teeth are lost is by the partial absorption of their roots, which, with coincident demolition of their sockets, leads to their being shed off, like a temporary tooth when its day is done, or like the practically devitalized tooth of the fish or reptile which is, after a brief sojourn, cast off and replaced by a new one.

This, as I shall presently endeavor to show, is one part of the problem: how to prevent a dead tooth from being shed.

It may be said that the argument goes to prove too much; that, like the absolutely unproved electrical theory of caries recently pressed so far, it provides for the certain destruction of all teeth in question; but this is not quite so. The recent conclusive researches of Heitzmann upon bone, extended by Bodecker to cementum, have shown that there is a protoplasmic net work occupying all the canaliculi and lacunæ of living bone and cementum, and that this protoplasmic network entering it from its surface, brings it into an intimate vital connection with the periosteum; in dead bone this has utterly disappeared. This protoplasmic network in the cementum becomes continuous at many points, through channels long since described with the protoplasmic fibers of the dentine: perhaps this connection may be of much practical significance.

A human tooth is therefore brought into continuity with the rest of the organism by two channels: by its alveolo-dental periosteum covering a great surface; and by its pulp constricted down to one or more small orifices; and these connections seem to bridge over the gap and render living parts tolerant of comparatively dead tissue. That the periosteum alone may be an adequate connection we know from daily experience of dead teeth successfully treated, teeth which have been knocked out and replaced at once, and teeth rendered practically pulpless by calcification of their pulps, though these are apt to be treated as foreign bodies and cast out by absorption of their roots.

I have recapitulated these anatomical and physiological facts—none of them new—because we need to have them clearly before our minds when considering the causes of failure.

I send round an upper molar which I treated unsuccessfully for weeks, and finally extracted; it exemplifies, in an extreme degree, a condition which I believe to be absolutely hopeless. When it was extracted it was merely rinsed in a basin of water, and never touched since. Everywhere the periosteum had ceased to be adherent to it, and it was, to all intents and purposes, an absolutely foreign body, held in by mere adaptation of its roots to their socket, but without a vestige of organic connection. It was, so to speak, a sequestrum: the protoplasmic network of its cementum was dead and gone, and, unless I mistake, in no way could such a tooth have been retained for any time. Of that tooth I can give you a complete history. Three years ago, though there was no exposure, I placed a little zinc oxychloride in the deepest portion of the cavity, and filled with amalgam over it. All went well for two years, when pulp irritation came on for no apparent reason. Failing to otherwise allay it, I devitalized with arsenic, experiencing some difficulty in doing so, owing to secondary dentine in the pulp. Contrary to my usual practice, I applied a further application of arsenic after the body of the pulp was dead, when it remained alive only in each of the three roots; the root pulps were afterwards most carefully and thoroughly removed. It was filled with creosote and wool in the roots, was never absolutely comfortable, was opened up and treated repeatedly, and at last, at the patient's desire, there being some neuralgia, extracted, although prior to its removal there was evidence merely of slight irritation in the socket. There was nothing to lead me to infer the complete detachment of the periosteum, and there was never at any time a vestige of pus formed. I am inclined to think that the arsenic may have destroyed not only the pulp, but have reached the protoplasmic network of the cementum. Doubtless we may often have to deal with partial death of this protoplasm, and may then sometimes succeed in retaining the tooth; death of the cement protoplasm on any considerable scale I believe to be an absolute bar to success.

I pass round another tooth—an upper latteral, the end of the root of which is eaten away in the most irregular way. Such teeth are not uncommon, and there is nothing remarkable about it, except that it was quite impossible to diagnose its condition; it looked a favorable case for ordinary treatment, and was extracted purely on account of the patient's inability to attend. Treatment must of course have signally failed, though replantation might have saved it for a year or two, and probably would have done so.

But in this tooth the state of things was very different. Here the apex was the thing affected, and the rest of the cementum with its protoplasm remained practically healthy; and here, so far as it has a point, lies the point of my communication.

I believe that in dealing with dead teeth we have two distinct conditions to combat; the one is abscess at the apex of the root, septic in its origin, and in its results leading, if it has time enough, sometimes to absorption, and sometimes to deposition on the root apices; and the other general disease of the periosteum, resultant upon disease or death of the cementum.

With apical abscess, taken in hand early, our percentage of success will be very high; when there has been time for change in the hard parts, it will be less complete, and the percentage lower; and here, if anywhere, is the legitimate field for replantation.*

Where we have a dead or dying cementum failure it seems to me is certain. Since I have thought on the matter in this light, two well-marked cases of necrosed cementum are all that I have seen. In each arsenic had been applied more than once, and left several days in the teeth.

There is much room for surmise, and still more for observation upon this matter. We do not know in the least what becomes of the protoplasmic contents of the dentinal tubes when a pulp is removed. Do they decompose and liquify, and, if so, may not their decomposition run on to the cement protoplasm with which they have many communications? Or may not the effect of arsenic travel along them in this direction, as we well know it used to in the other (*i. e.*, towards the pulp) when it was formerly used to allay the sensitiveness of dentine.

If so, we should be very careful to minimise the time of its ap-application, and scrupulously keep it out of roots.

Or, perhaps the dentinal fibrils may not decompose, but may keep up some feeble organic life through their inosculation with the cementum protoplasm. In such a case, should we do well to fill the roots off-hand the moment we have removed the remnant of living nerve from the pulp cavity? For it is not to be forgotten that some years ago many practitioners used to do this, and even fill with gold, which now we should do, after days or weeks of creosote treatment, with fear and hesitation.

To recapitulate. I believe that in the treatment of dead teeth we have to combat not only the troubles which arise from the escape of septic matter from the apex of the root, which everybody recognizes, and in a way understands, but also disease of the alveolo-dental periosteum, induced in some other way, which is less frequent but more intractable. If the surmise that the alveolo-dental periosteum does become diseased in other ways than

* In these remarks I am of course speaking only of teeth the roots of which are large enough to enable us to do all that we wish to our satisfaction; I am leaving out of course the failures due to crooked and impervious roots, in other words to imperfect operations.

by the escape of septic material from the apical root canal be true, it is difficult to see by what channel evil influence may approach it other than by the protoplasm of the cementum. This again would hardly be reached except through the medium of the contents of the dentine tubes in the dentine of the root. Such an evil influence may, perhaps, be septic; *i. e*, the putrefaction of the soft parts of the dentine may poison those of the cementum, and this would seem to be the most likely thing; or the destructive influence of such agent as arsenic may be propagated through the same tissues and bring about similar results in the cementum. In either case we shall have to look to the dentine of the root as the proximate cause of the mischief.

In the absence of adequate positive knowledge, conjecture may sometimes play a useful part. This must be my apology for my imperfect and inconclusive communication.

I have sometimes employed shellac for filling roots which are so fine as to render it very difficult or impossible to fill them with the materials ordinarily in use.

If it is drawn out into fine threads it is flexible and elastic, and will pass anywhere where the very finest hair bristle will pass; at the same time it is brittle enough to break off readily without withdrawal, and so soon as no more can be introduced by the side of that already in, a drop of spirit running up by capillary attraction fixes it and converts it into a very perfect filling even in very small and curved roots. It possesses this one advantage over all other root-filling materials with which I am practically acquinted: it can be introduced even into the finest root and a perfect filling made without any danger of a piston being formed to force out septic matter from the end of the root.

A little patience and hot instruments will get it out again from a large root, and I have had to thus remove it, but it would be all but impossible to get it out of a very narrow canal, and hence its use is limited to those cases in which one feels tolerably sure of not having irritation follow the filling. This same objection, however, applies to all perfect fillings in minute roots. I leave wool, of course, out of the question, as it is simply impossible to get it up such roots; but with shellac roots can be perfectly filled which can not be filled with any other material with which I am acquainted, not excepting gold wire, as the threads of shellac are easily made of a degree of fineness not ready attainable with a very soft gold wire.

